# Nurses as Stakeholders in the Adoption of Mobile Technology in Australian Health Care Environments: Interview Study

**DOI:** 10.2196/14279

**Published:** 2019-08-09

**Authors:** Carey Mather, Elizabeth Cummings, Fred Gale

**Affiliations:** 1 College of Health and Medicine School of Nursing University of Tasmania Launceston Australia; 2 School of Health Information Science University of Victoria Victoria, BC Canada; 3 College of Arts, Law and Education School of Social Sciences University of Tasmania Launceston Australia

**Keywords:** digital, health policy, digital professionalism, nursing education

## Abstract

**Background:**

The 2017 Australian Digital Health Agency (ADHA) Strategy is based on the underlying assumption that digital technology in health care environments is ubiquitous. The ADHA Strategy views health professionals, especially nurses, as grappling with the complexity of installing and using digital technologies to facilitate personalized and sustainable person-centered care. Yet, ironically, the 2018 debate over how to enroll Australians into the national electronic health record system and its alteration from an opt-in to an opt-out model heightened public and professional concern over what constituted a “safe, seamless and secure” health information system. What can be termed a digital technology paradox has emerged where, although it is widely acknowledged that there are benefits from deploying and using digital technology in the workplace, the perception of risk renders it unavailable or inaccessible at point of care. The inability of nurses to legitimately access and use mobile technology is impeding the diffusion of digital technology in Australian health care environments and undermining the 2017 ADHA Strategy.

**Objective:**

This study explored the nature and scope of usability of mobile technology at point of care, in order to understand how current governance structures impacted on access and use of digital technology from an organizational perspective.

**Methods:**

Individual semistructured interviews were conducted with 6 representatives from professional nursing organizations. A total of 10 interview questions focused on factors that impacted the use of mobile technology for learning at point of care. Seven national organizations and 52 members from the Coalition of National Nursing and Midwifery Organisations were invited to participate. Interviews were recorded and transcribed verbatim. Data analysis was systematic and organized, consisting of trial coding; member checking was undertaken to ensure rigor. A codebook was developed to provide a framework for analysis to identify the themes latent in the transcribed data. Nurses as stakeholders emerged as a key theme.

**Results:**

Out of 6 participants, 4 female (67%) and 2 male (33%) senior members of the nursing profession were interviewed. Each interview lasted between 17 and 54 minutes, which reflected the knowledge of participants regarding the topic of interest and their availability. Two subthemes, coded as *ways of thinking* and *ways of acting*, emerged from the open codes. Participants provided examples of the factors that impacted the capacity of nurses to adopt digital technology from an emic perspective. There were contributing factors that related to actions, including work-arounds, attentiveness, and experiences. Nurses also indicated that there were attitudes and influences that impacted thinking regarding access and use of mobile technology at point of care.

**Conclusions:**

Nurses are inadequately prepared for the digital future that has now arrived in health care environments. Nurses do not perceive that they are leaders in decision making regarding digital technology adoption, nor are they able to facilitate digital literacy or model digital professionalism.

## Introduction

The rapid evolution of health technology and informatics has significantly altered health care delivery and impacted the health care workforce in Australia and internationally [1-4]. In Australia, nurses are the largest group of registered health professionals [5]; however, as digital technology has been introduced into health care environments, nurses have struggled to be included in decision-making processes [6]. A failure to provide clear direction to nurses about access to, and use of, digital technology at systems, organizational, and individual levels has been found to be a contributing factor to disempowering nurses [7]. Additionally, lack of empowerment can also be attributed to the cost of preparing nurses to become digitally literate and capable of enabling other stakeholders, such as consumers, to become proficient end users of digital technology [8]. Fear of inappropriate use [9,10] and resistance to changing workflow routines [11] also play a role in the lack of agency that nurses exhibit in advocating for the adoption of digital technology to advance nursing practice.

Increasing awareness of the need for change is evident in a number of initiatives. The release of the Australian Digital Health Agency (ADHA) Strategy [12] outlined seven strategic priorities, with the sixth one highlighting the importance of workforce education and training of health professionals. The ADHA Strategy underpins changes being implemented by the Australian Nursing and Midwifery Accreditation Council (ANMAC) in its review of standards for accrediting undergraduate nursing education programs. Previously, an explanatory note [13] was published to clarify the expectation of health technology and health informatics to be included at a technical, contextual, and emancipatory level into new nursing curricula. More recently, ANMAC has proposed that the integration of health technology and health informatics be articulated more prescriptively in the revised standards [14,15]. Clear direction regarding the required minimum standard of capability of undergraduate students to be work ready at registration will create pressure on organizations and higher education institutions to initiate or further promote preparation of the nursing workforce to be digitally literate and digitally professional [7,16]. There will be a need to accommodate the impending changes within curricula that will impact work-integrated learning.

Other recent initiatives include the development of national nursing informatics competency standards to provide guidance to nurses about the expected level of understanding of computer and information literacy and management [17]. Another includes the release of the combined Australian nursing and health informatics organizations’ Health Informatics Position Statement [18] outlining professional expectations of all stakeholders of health technology and health informatics implementation in health care settings. Outputs prioritized by the ADHA Strategy [12] include the following, in order to support health professionals currently employed within health care settings: “resources and curricula will be developed to ensure healthcare practitioners are exposed and trained in digital technologies and their use during training and upskilling.” Furthermore, the employment of clinical informatics champions as outlined in the strategy will drive cultural change and awareness at a local level. Concurrently with the release of the ADHA Strategy [12] was a change to the rollout of the national electronic health record—My Health Record—from an opt-in to an opt-out system for all Australian citizens. This process was precipitated by a range of factors, including reluctant voluntary uptake due to numerous data privacy and security breaches [19,20]. Currently, less than 25% of all Australian citizens have an electronic health record [21], although this is now expected to expand dramatically. However, given that there had been inadequate training for frontline health professionals, including nurses, to improve their digital literacy or educational preparation, they will continue to struggle to explain to consumers the merit of having their own digital health record [22].

The release of the Nursing Informatics Position Statement [18] demonstrates that nurses within the health technology and health informatics field recognized the pivotal role of nurses to successfully implement digital technologies within health care environments. The position statement articulated seven elements that outlined the need for a strong nursing presence in governance and decision making at systems, organizational, and individual levels to “safeguard adoption and optimization of clinical information systems” [18]. It was in this broad context of structural change within the Australian health technology and health informatics field that this research examined the use of mobile technology for informal learning and continuing professional development (CPD) of nurses at point of care. The research aimed to understand the factors influencing mobile technology policy development from the perspective of nursing profession organizations. The purpose of this study is to inform the profession about the current status of using digital technology at point of care so that the nursing profession can advocate its perspective at a national level and become more included in policy decisions affecting the nursing profession.

## Methods

### Recruitment

Individual semistructured interviews were undertaken with 6 representatives from professional nursing organizations. Purposive sampling was used to recruit participants who were able to represent organizations from a policy or guideline perspective and who had expertise in nursing practice. Seven national organizations were identified and a further 52 members from the Coalition of National Nursing and Midwifery Organisations (CoNNMO), who had email addresses available on their website, were invited to participate. Follow-up emails 2 weeks after the initial invitation and a reminder email were sent 1 month after the first invitation. Those organizations that listed telephone details were also contacted via telephone. An information sheet was provided with the invitation to participate, and consent was recorded using Skype for Business prior to the beginning of each participant interview. Participants chose the venue and time for the interview. The University of Tasmania Social Sciences Human Research Ethics Committee granted approval (approval number: H0016097) prior to initiating this study.

### Interview Schedule

A total of 10 interview questions were developed (see Multimedia Appendix 1) from the findings of previous research [23] and focused on whether the nursing profession organizations had a policy position on mobile technology for informal learning and CPD. Questions then explored factors impacting the use of mobile technology for learning at point of care. Interviews were undertaken by the first author (CM) during December 2016 and January 2017 and transcribed verbatim.

### Data Analysis

**Figure 1:**
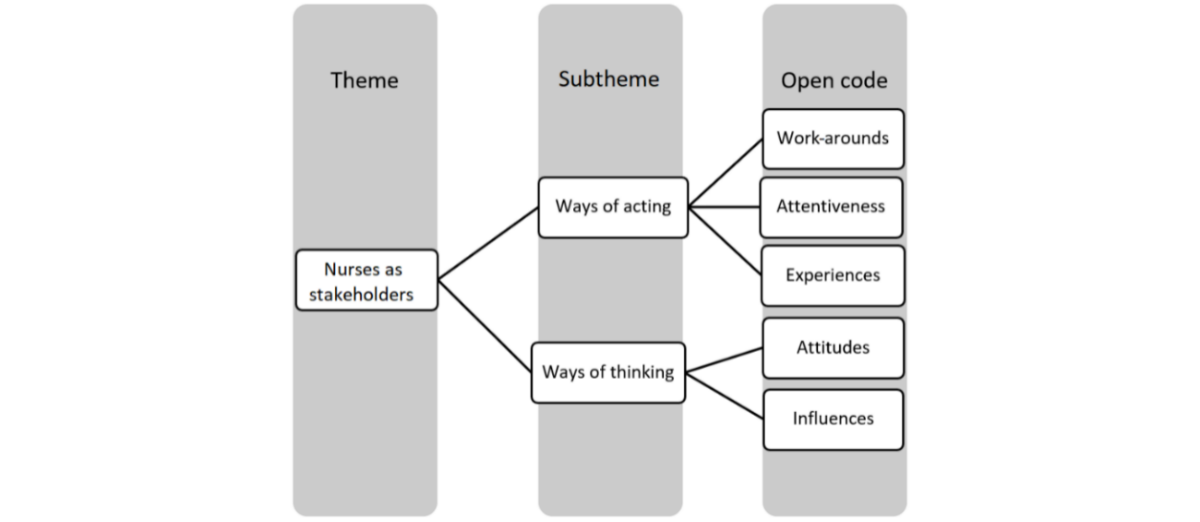
Coding process.

Data analysis was a systematic and organized process consisting of trial coding; constant member checking was undertaken to ensure rigor. A codebook was developed to provide a framework of codes. Fidelity of application of labels across interviews to ensure consistency was undertaken during coding. Microsoft Excel 2016 was used to tabulate *meaning units* [24]. Reducing the phrases by coding enabled further refinement, and the subthemes of *ways of acting* and *ways of thinking* were emergent from the data. From the analysis, *nurses as stakeholders* was identified as a theme (see Figure 1).

## Results

### Overview

Six interviews were conducted during the data collection period. Of the 6 participants, 4 were female (67%) and 2 were male (33%). All participants identified themselves as senior members of the nursing profession with extensive clinical, educational, research, or administrative experience in a range of health care environments (see Table 1).

These nurses were paid employees or were associated with CoNNMO as volunteers within Australian nursing specialty professional bodies. Each interview lasted between 17 and 54 minutes. The length of each interview was related to each participant’s available time and knowledge regarding the topic of interest. Two subthemes coded as *ways of acting* and *ways of thinking* emerged from the open codes (see Figure 1).

**Table 1:** Participant demographics.

Interview #	Gender	Source of recruitment	Nursing organization	Nurse role
1	Female	Direct email to organization	National representative (executive)	Administration
2	Male	Email from CoNNMO^a^ secretariat	Specialty nursing executive position (volunteer organization)	University academic and clinician
3	Female	Direct email to organization	National representative (executive)	University academic
4	Female	Email from CoNNMO secretariat	Specialty nursing executive position (volunteer organization)	Clinician
5	Male	Email from CoNNMO secretariat	Specialty nursing executive position (volunteer organization)	Administration and clinician
6	Female	Direct email	National representative (executive)	Administration

^a^CoNNMO: Coalition of National Nursing and Midwifery Organisations.

### Ways of Acting

#### Overview

Participants provided examples of how nurses behaved in relation to accessing and using digital technology in the workplace. These nurses highlighted the risks, challenges, barriers, and benefits of accessing and using digital technology in health care service provision and for learning and teaching. Participants indicated that the capacity for digital technology adoption by nurses was affected by contributing factors related to actions linked to work-arounds, attentiveness, and experiences. Each is explained in detail below.

#### Work-arounds

When listening to and coding their conversations, it became apparent that participants often perceived that they did not have a voice in decision making regarding access and use of digital technology. The lack of inclusion resulted in unintended consequences. Nurses developed work-arounds to accommodate perceived workflow issues [25]. For example, one participant indicated that nurses used their personal mobile devices to seek and retrieve information:

I did a survey of about 10 [city] hospitals, [specialty] departments, and less than half the staff had Internet access and when you take out the senior staff in that survey, there was, you know, most of the staff, the direct care nurses didn't have Internet access. So, people are using their mobile phones for Internet access and to find evidence and to source, you know, information, which is just terrible.

Participant #2

Another participant stated that due to funding constraints, using a mobile device enabled improved efficiency of health care service delivery by facilitating access to information:

You know, they’re taking away massive amounts of funding and things are very privatized, so these facilities are kind of left to their own devices as far as how many RNs [registered nurses], if they have RNs at all now. So, I think that one way—and I don’t know how to combat that—but one way to ensure better patient care is by allowing staff to have that kind of information where they can look at a patient and put symptoms or use education that’s right there and then on their phones and that will probably lead to better patient care and identification of deterioration or issues. I think that’s an area that could be incredibly useful.

Participant #4

#### Attentiveness

There were divergent views about accessing digital technology in relation to attentiveness. Participants provided examples of how mobile devices can be used in real time for improving efficiency of health care delivery. For example, one participant outlined a benefit of integrating digital technology into nursing practice:

But if you’re actually learning in real time as you’ve got an actual issue happening, it’s great. I think there’s also potential to use downtime better. I was going to say like everything that’s mobile, it just becomes integrated more into people’s lifestyle.

Participant #6

However, there was also the view that there needed to be a minimum standard of capability of nurses rather than relying on digital technology when there was the potential to negatively impact safe health care delivery:

...you know, you’re not going to be sitting there in the middle of a [cardiac] arrest going, “Okay, wow, the doctor’s just asked for adrenalin, hold on let me just quickly Google adrenalin and find out how fast I should push it and is the actual correct dose and, oh, what’s its indication, oh, what’s its mode of action?”

Participant #5

Another participant indicated that there were perceived risks associated with distraction when using mobile technology:

People will get enamored or caught up on what's going on in their phone and not be paying attention to what's going on with their patient.

Participant #1

#### Experiences

Nurses provided examples of how digital technology could change behavior to enhance nursing practice and support learning by students while undertaking work-integrated learning:

Particularly, I worked in rural and remote-type areas so where you didn’t have someone else that you could ask for help, and just for the students I've found that if they can look something up then and there, that they learn it because it makes sense to them, that they need to know it.

Participant #3

Additionally, participants were aware that the digital revolution was imminent. They could foresee the benefits of enabling personalized learning that could contribute to supporting person-centered care. One participant stated the following:

I think it’s got huge potential for really looking at being able to adapt to different learning styles and being able to bring that, the ideal situation where you’ve got theory with experience at the same time, and you can look at what you’ve assessed and then put that into mobile learning technology and find out what you’ve missed or how that measured up against the theory on the information.

Participant #4

Participants acknowledged that the role of nurses included experiences that promoted engagement and improvement in health literacy, digital literacy, and health education of consumers. One participant indicated the following:

I think too that a lot more consumers are going to the Internet for information these days; to be able to show them what is a useful site or is a safe site for them to go to so they're not getting false information about things would be quite useful as well. So, I think there's significant benefits there.

Participant #3

### Ways of Thinking

#### Overview

The second subtheme that emerged was *ways of thinking*. Nurses in this study provided their perceptions of what nurses thought from an emic perspective and how this impacted the use of digital technology in health care settings. Participants provided rich descriptions about the attitudes and influences—the two open codes associated with this subtheme that they encountered—in relation to digital technology and the nursing profession.

#### Attitudes

Nurses raised the issue of balancing person-centered health care delivery, learning, and integrating digital technology within nursing practice. One participant stated the following:

I think there's an issue on duty in that the staff will say they've got enough to do without having to sit down at the computer...if they are allocated time in their workday to do it, well then that's probably okay. But if they're just expected to fit it on around everything else, I'm not sure how much focus they'll give it.

Participant #1

However, participants also acknowledged there was a range of attitudes about nurses using digital technology in health care environments. An example quotation about nurses being viewed negatively by other stakeholders is shown below:

...all the nurses seem to do is pay attention to the computer even where they've got electronic health records and order entry systems and all that they have now. And you do hear at times members of the community see that the nurses are tied more to the computers than they are tied to the people.

Participant #1

In stark contrast, a participant whose attitude was positive regarding implementation of digital technology into nursing practice stated the following:

We're just burying our head in the sand saying, you know, let's just say no phones, well that's not happening. Anyway, it's actually detrimental because it's a really useful tool, these mobile devices, for our staff. We can just train people better in how to use their phone.

Participant #2

#### Influences

Implementation of digital technology will require support at systems and organizational levels before stakeholders will be influenced to adopt it to advance nursing practice. One participant indicated the following:

So, I think there’s a lot going on but there’s not—as far as I’m aware—there’s not a really big push from the government to use technology, or funding from the government to use technology well. And there’s lots of, I guess, private and smaller initiatives taking off, but I think the drive has to be from the health district as well, but there has to be government funding and incentives to be implementing technology and, particularly, mobile technology.

Participant #4

At an individual level, one participant indicated that nurses have the capacity to change their views when exposed to the benefits of using digital technology. However, others are also influenced, which impacts on nurses’ capacity to use digital technology within their workplace. The following quotation illustrates this view:

I've certainly had clinical facilitators in the past make comments that, you know, it has been changing and that they'd seen students with mobile technology and gone to them with the idea of criticizing them for using it and then discovered that they were using it for very relevant purposes. And their attitudes were changing due to that type of thing, so I'd been supportive of that type of learning and the success they were having. But now it's very different, we’ve got an edict from above and we're not allowed to do it [use digital technology].

Participant #3

## Discussion

### Principal Findings

Findings from this research indicate that nurses within the profession’s organizations do not perceive that they are leaders in decision making regarding digital technology. Participants believed that nurses’ access to, and use of, the Internet within health care environments is decided by others. This perception shapes their ways of acting and thinking, which impacts on their capacity to advocate for being included as stakeholders regarding health technology and health informatics at systems, organizational, and individual levels.

Participants realize that there is a mobile learning paradox where, although it is acknowledged that there are benefits to using digital technology, they are unable to access it [26]. This paradox extends beyond using mobile technology at point of care. Participants acknowledged that work-arounds have occurred to accommodate the lack of access to the Internet by individual nurses providing direct care to patients. Additionally, nurses deployed digital technology within their nursing practice to improve efficiency due to funding constraints. Some participants lamented the lack of capacity to harness the benefits of digital technology, while others understood the risks of digital technology being used inappropriately. These views are congruent and support the efforts being made by ANMAC to ensure that undergraduate nurses become digitally professional and work ready at graduation [14,15]. Furthermore, the ADHA Strategy will provide support for CPD of nurses currently working within health care environments and will underpin the foundational knowledge of undergraduate students [16]. A period of overlap will be required as registered and student nurses learn from each other and develop a mutual understanding [27] of the knowledge and skills required to meet the National Informatics Standards [17]. There is potential for the development of agency by nurses when they share their experiences in this way and, as demonstrated by the findings, nurses are capable of adapting their behavior based on their experiences.

When undergraduate nurses are educationally prepared to use health technology and health informatics, and to undertake work-integrated learning, they will be better able to challenge the status quo arrangements that marginalize them and to request inclusion in accessing and using digital technology. For example, registered nurses are permitted password access, whereas in the future, supervising nurses and undergraduate students conversant with policy and guideline documents [12,17] will be able to lobby health care organizations for password access to health information systems for documentation purposes. Students will also be keen to translate their learning about patient information flows within a simulated environment by documenting nursing activities in an operational electronic health record while in practice. Additionally, students indicated that they prefer to access learning resources in real time at point of care [28]. Personalized learning and the development of digital literacy by students early in their studies will benefit all stakeholders, including consumers, who can be influenced by nurses to participate in their own care when the nurses are confident and capable in using digital technology.

This research supports the need for a digital health strategy as proposed by the ADHA [12]. However, to enable adoption, there is a need for nurses to become leaders by ensuring that they are involved in decision making regarding implementation of health technology and informatics in health care environments.

**Figure 2:**
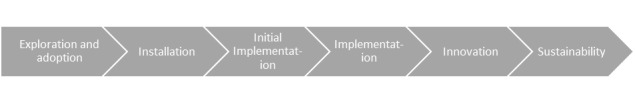
Stages of implementation (modified from Fixsen et al, 2005).

Fixsen and colleagues [29] identified six stages of implementation (see Figure 2) that can be applied to the evolution of digital technology within the Australian health care context. Exploration and adoption of digital technology has been followed by trial installations and initial implementation of various applications and health information systems. The publication of the ADHA Strategy’s strategic priorities [12] demonstrates that the Australian health care sector is now positioned for full implementation of health technology and informatics (see Figure 2). However, there have been delays in deploying health information systems within some Australian states. Western Australia and Queensland health care providers have experienced data systems failures that have reduced citizen trust and delayed implementation of electronic record systems [19,30]. Such mistrust could be partially remediated by nurses being more included in organizational decision making [31], given that nurses are the largest group of stakeholders within the health care sector [5]. This research highlights the urgent need for this group of health professionals to be fully engaged in the digital future of health care environments.

### Limitations

Limitations of this study include the timing of the interview period, which spanned the traditional end-of-year Christmas and summer holiday period; this may have contributed to the low participation rate. Interviews ceased with the publication of the Nursing Informatics Position Statement [18], as the researchers considered that this statement had the potential to influence the nursing profession organizations’ views about health informatics. The low participation rate reduces generalizability of the findings.

### Future Directions

A larger international comparative study could be undertaken to replicate this research. Findings may determine whether registered nurses in other countries experience similar perceptions or whether the Australian context is unique.

### Conclusions

There is still much work to be undertaken to engage all stakeholders, including nurses, in embracing the digital future in health care. This research demonstrates that nurses from professional organizations understand their health workforce but lack the agency to demand inclusion in decision making that impacts nurses at organizational and individual levels. To enable implementation of health technology and informatics in health care environments more effectively, it is crucial for nurses to become stakeholders at every level. Doing so will not only mitigate the risk of implementation failure, but engagement of nurses as frontline health professionals will assist the Australian Government in achieving its goal of a “safe, seamless and secure” digital health system for all.

## Appendix

Multimedia Appendix 1 Nursing profession organization interview schedule.

## Abbreviations

ADHA: Australian Digital Health Agency
ANMAC: Australian Nursing and Midwifery Accreditation Council
CoNNMO: Coalition of National Nursing and Midwifery Organisations
CPD: continuing professional development
RN: registered nurse
