# Supplementary material for: Nurses as Stakeholders in the Adoption of Mobile Technology in Australian Health Care Environments: Interview Study
Source: JMIR Nurs. 2019 Aug 9;2(1):e14279. doi: 10.2196/14279 (PMC8279446; doi:10.2196/14279)
Supplement: Multimedia Appendix 1 [file nursing_v2i1e14279_app1.docx]

#### Nursing profession organisation interview schedule:

#### Exploring influencing factors on policy development in Australia

| **Overview of organisations’ position on mobile technology** |  |
| --- | --- |
| **Key Questions** | **Funnelling Question** |
| 1. Can you tell me about the overall view of this organisation’s position on nurses and midwives using mobile technology for informal learning and CPD in the workplace? |  |
| 2. If your organisation has a position on mobile technology use for mobile learning, please provide detail about how this position was developed? | a. History  b. Process undertaken  c. Documents used |
| 3. If your organisation has no position on mobile technology use for mobile learning, what do you think this organisation could offer in order to influence the use of mobile technology for informal learning and CPD in the workplace? | a. Positives  b. Negatives |
| 4. Can you tell me what your organisation can do to support the development of standards, guidelines or policies about the access and use of mobile technology at point of care? | a. Positives  b. Negatives |
| **Mobile technology and learning** |  |
| 5. Now I’d like to move on to talk about the potential of learning using portable or mobile technology in the workplace. Can you explain to me in your own words how portable or mobile technology could change learning in the workplace? |  |
| 6. Can you tell me about how your organisation’s opinion on access to portable or mobile learning environments impact on patient or client safety? | a. Positives  b. Negatives |
| 7. Can you tell me about your organisation’s opinion on perceptions of public about nurses and/or midwives using portable or mobile technology in the workplace? | a. Positives  b. Negatives |
| 8. Do you have any opinion on perceptions of other health professionals using portable or mobile technology in the workplace? | a. Positives  b. Negatives |
| **Continuing professional development** |  |
| 9. To finish up I want to ask a question about nurses or midwives using mobile technology for informal learning or continuing professional development more broadly.  What do you perceive nurses or midwives currently do for continuing professional development to meet the requirements for AHPRA? | a. If they use web-based resources can you give examples of what you know can be accessed or used?  b. What is your opinion of using mobile learning to achieve some of the CPD requirements? |
| 10. Do You have any other comments you would like to make regarding nurses using mobile technology for learning? |  |
